# The Hepatic *Raldh1* Expression Is elevated in Zucker Fatty Rats and Its Over-Expression Introduced the Retinal-Induced *Srebp-1c* Expression in INS-1 Cells

**DOI:** 10.1371/journal.pone.0045210

**Published:** 2012-09-13

**Authors:** Yang Li, Yan Zhang, Rui Li, Wei Chen, Meredith Howell, Rui Zhang, Guoxun Chen

**Affiliations:** Department of Nutrition, the University of Tennessee at Knoxville, Knoxville, Tennessee, United States of America; National Institutes of Health, United States of America

## Abstract

The roles of vitamin A (VA) in the development of metabolic diseases remain unanswered. We have reported that retinoids synergized with insulin to induce the expression of sterol-regulatory element-binding protein 1c gene (*Srebp-1c*) expression in primary rat hepatocytes. Additionally, the hepatic *Srebp-1c* expression is elevated in Zucker fatty (ZF) rats, and reduced in those fed a VA deficient diet. VA is metabolized to retinoic acid (RA) for regulating gene expression. We hypothesized that the expression of RA production enzymes contributes to the regulation of the hepatic *Srebp-1c* expression. Therefore, we analyzed their expression levels in Zucker lean (ZL) and ZF rats. The mRNA levels of retinaldehyde dehydrogenase family 1 gene (*Raldh1*) were found to be higher in the isolated and cultured primary hepatocytes from ZF rats than that from ZL rats. The RALDH1 protein level was elevated in the liver of ZF rats. Retinol and retinal dose- and time-dependently induced the expression of RA responsive *Cyp26a1* gene in hepatocytes and hepatoma cells. INS-1 cells were identified as an ideal tool to study the effects of RA production on the regulation of gene expression because only RA, but not retinal, induced S*rebp-1c* mRNA expression in them. Recombinant adenovirus containing rat *Raldh1* cDNA was made and used to infect INS-1 cells. The over-expression of RALDH1 introduced the retinal-mediated induction of *Srebp-1c* expression in INS-1 cells. We conclude that the expression levels of the enzymes for RA production may contribute to the regulation of RA responsive genes, and determine the responses of the cells to retinoid treatments. The elevated hepatic expression of *Raldh1* in ZF rats may cause the excessive RA production from retinol, and in turn, result in higher *Srebp-1c* expression. This excessive RA production may be one of the factors contributing to the elevated lipogenesis in the liver of ZF rats.

## Introduction

The high obesity prevalence in the population of the United States [1] predicts the increase of patients with noninsulin-dependent diabetes mellitus (NIDDM) [2], a major public health concern [3]. Genetic mutations, such as mutations of leptin and its receptor, have been shown to cause the development of obesity and diabetes [4]. Currently, the associations of a variety of genes with the development of human obesity or NIDDM have been indicated [5], [6]. Metabolic abnormalities are often associated with profound changes of hepatic glucose and lipid metabolism [7], which is attributed, at least in part, to the expression of genes involved in these processes [8], [9]. On the other hand, overconsumption of nutrients, such as fructose in sweetened beverages, has also been implied to play a role in the rise of obesity [10]. Dietary nutrients provide us with not only energy, but also vitamins and other essential factors with regulatory roles. The effects of individual micronutrients on the development of metabolic diseases remain to be revealed.

As an essential and fat-soluble micronutrient, vitamin A (VA, retinol) plays crucial roles in the general health of an individual, such as vision, tissue differentiation, immunity, etc [11]. The majority of the physiological actions of retinol are mediated by its active metabolite, retinoic acid (RA), which exists in multiple isomeric forms, such as all-*trans* RA and 9-*cis* RA [12]. RA regulates gene expression through the activation of two families of nuclear receptors, retinoic acid receptors (RARα, β and γ) activated by all-*trans* RA, and retinoid X receptors (RXRα, β and γ) activated by 9-*cis* RA [13].

In an attempt to understand the effects of endogenous lipophilic molecules on the insulin-regulated gene expression in primary rat hepatocytes, we have analyzed the rat liver lipophilic extract and found that retinol and retinal in it dose-dependently induced the mRNA expression levels of *Pck1* [14], *Gck* [15], and *Srebp-1c* [16]. The proximal one of the two previously identified retinoic acid response elements (RAREs) in *Pck1* promoter [17]–[19] mediates the RA effect in primary hepatocytes. The previously identified two liver X receptor (LXR) binding sites mediating the insulin-stimulated *Srebp-1c* expression [20] were also RAREs in its promoter [16], demonstrating the convergence of hormonal and nutritional responses. The hepatic expression level of cannabinoid receptor 1 (CB1), the target of anti-obesity drug rimonabant [21] can also be induced by the treatment of RA via activation of RAR-γ [22], indicating the connection between the nutritional signals and endocannabinoid pathways. The effects of VA status on glucose and lipid metabolism have been summarized [23].

Retinol is reversibly oxidized into retinal by retinol dehydrogenases (RDHs). Retinal is irreversibly oxidized into RA by retinaldehyde (aldehyde) dehydrogenases (RALDHs) [13], [24], [25]. Currently, four RALDHs have been cloned and thought to be responsible for all-*trans* or 9-*cis* RA production in various tissues [26]–[29]. RALDH1-4 proteins can be detected in some but not all hepatocytes of the mouse liver using immunohistochemistry, with RALDH1 being frequently expressed in lipid-engorged cells [27]. *Raldh1* (also known as *Aldh1a1*) mRNA is expressed weakly in the rat liver [26], whereas *Raldh4* (also known as *Aldh8a1*) mRNA is expressed at high level in the mouse liver [27]. The mice with *Raldh1* deletion (*Raldh1*−/−) have reduced RA production in the liver two hours after a challenge of retinol [30]. The *Raldh1*−/− mice are resistant to diet-induced obesity (DIO) and insulin resistance [31]. The Imprinting Control Region (ICR) mice fed a high cholesterol diet had the elevation of the hepatic expression of RALDH1 and RALDH2 through oxysterol-induced *Srebp-1c* expression, demonstrating the interaction of cholesterol and RA metabolism [32]. All these observations indicate the potential roles of these enzymes in the development of obesity and insulin resistance, which deserves to be further defined.

**Figure 1:**
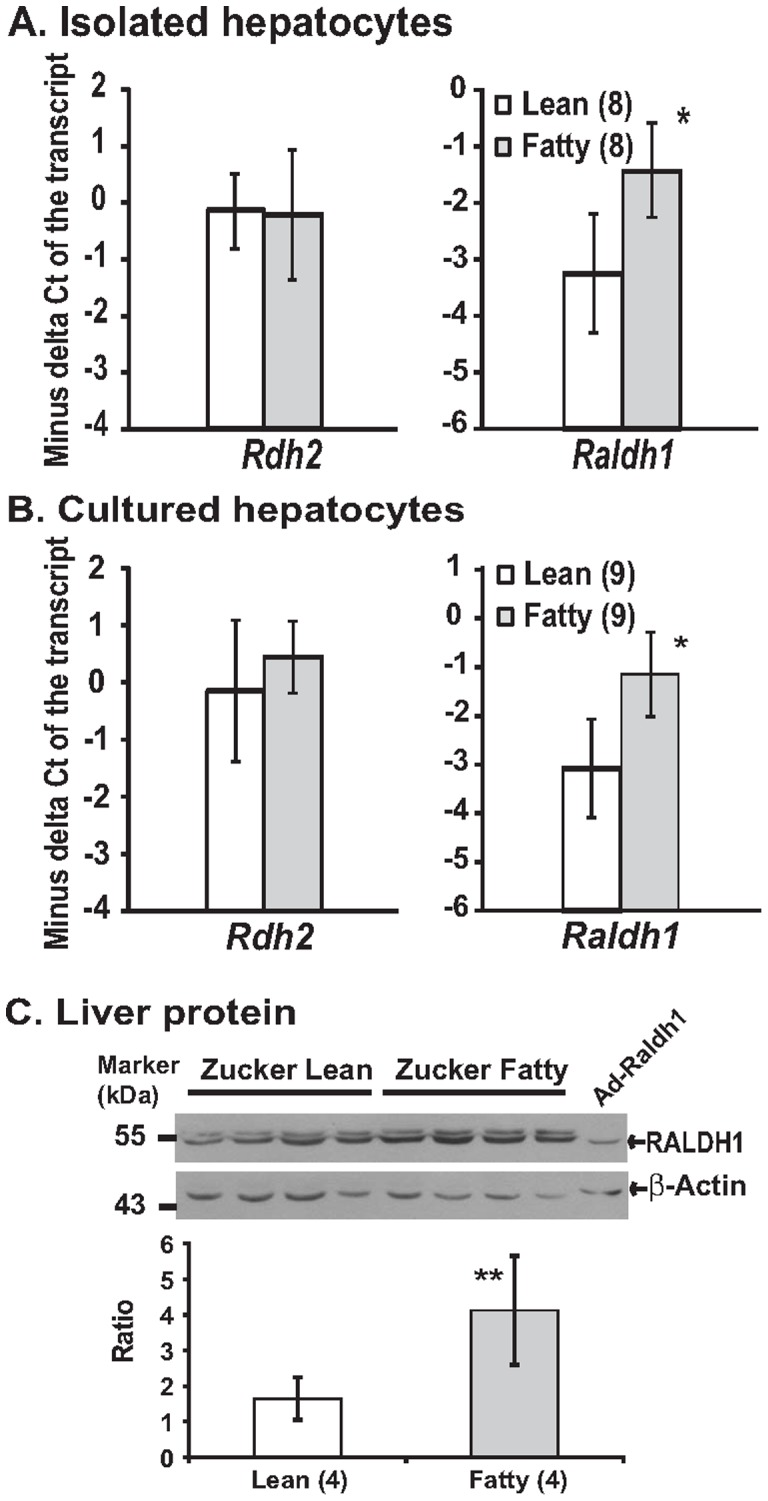
The mRNA levels of *Rdh2,* and *Raldh1* in freshly isolated (A) and cultured (B) primary hepatocytes, and the RALDH1 protein levels (C) in the liver of *ad libitum* ZL and ZF rats. **A and B.** Total RNA was extracted from hepatocytes and subjected to real-time PCR analysis. Results were presented as means ± SD of the −ΔC_T_ (the C_T_ of 36B4– the C_T_ of indicated transcript) from the indicated different numbers (in parenthesis) of hepatocyte isolations (* all *p*<0.05, for comparing the values of ZL and ZF groups of the indicated transcripts using the independent t-test). **C.** Whole liver tissue lyastes (100 µg/lane) of ZL (lanes 1–4) and ZF (lanes 5–8) rats, and whole cell lysate of INS-1 cells infected by Ad-Raldh1 (50 µg, lane 9) were separated in 10% SDS PAGE gels, and transferred to the PVDF membranes as described in the Material and Methods. Primary antibodies to RALDH1 (1∶1000 dilution in TBST containing 5% dry milk), and to β-Actin (1∶1000 dilution in TBST containing 5% bovine serum albumin) were recognized by goat anti-rabbit IgG conjugated to horseradish peroxidase, and visualized by chemiluminescence. The ratios of RALDH1/β-Actin were calculated after the films were scanned and analyzed. Data were presented as mean ± SD of the indicated numbers (in parenthesis) of ZL and ZF rats (** *p*<0.04 for comparing the ratios of ZL rats with that of ZF rats using the independent t-test).

The fact that the levels of the enzymes for the hepatic retinoid metabolism can be dynamically regulated leads us to hypothesize that the change of the expression of these enzymes and the production of RA may contribute to the regulation of the expression of hepatic lipogenic genes. Here, we examined the effects of retinol and retinal on the expression of RA responsive genes in primary rat hepatocytes, rat hepatoma cells and insulinoma INS-1 cells. We observed the elevated expression levels of *Raldh1* mRNA in hepatocytes and its protein in the liver of Zucker fatty (ZF) rats. The recombinant adenovirus-mediated over-expression of RALDH1 resulted in retinal-mediated induction of *Srebp-1c* expression in INS-1 cells, which originally lack the response to retinal.

**Figure 2:**
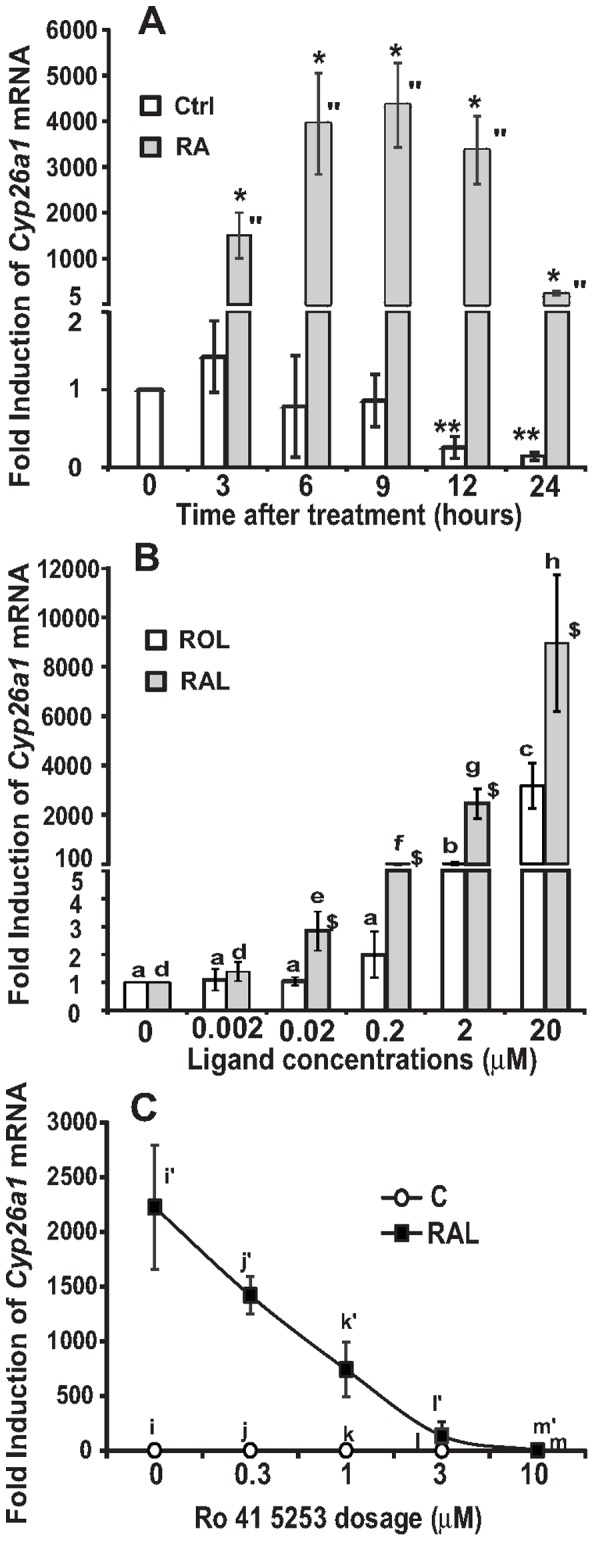
The expression levels of *Cyp26a1* mRNA in response to RA treatment over a 24-h time period (A), to different dosages of ROL or RAL (B), and to an antagonist of the RAR activation (C). Primary hepatocytes were isolated from SD rats, and pre-treated as described in the Materials and Methods. Total RNA was isolated, and subjected to real-time PCR analysis. **A.** A 24-h time course of *Cyp26a1* mRNA expression induced by RA (5 µM) in primary rat hepatocytes (* for comparing the vehicle control with the RA groups at the indicated time points using the independent t-test; ** and '' for comparing the different time points of vehicle control or RA group using one way ANOVA; all *p*<0.05). **B.** The expression levels of *Cyp26a1* mRNA in primary hepatocytes treated with increasing concentrations of ROL or RAL for 6 hours ($ for comparing the fold induction of ROL group with that of RAL group at the indicated concentrations using the independent t-test; c > b > a; h > g > f > e > d for comparing the different dosages of ROL or RAL using one way ANOVA; all *p*<0.05). **C.** Inhibition of the RAR activation disrupted the induction of *Cyp26a1* by RAL treatment. Primary hepatocytes were incubated in medium A without or with 2 μM RAL in the absence or presence of increasing concentrations of Ro 41–5253 for 6 hours (i/j > l, i'/j' > k' > l' > m' for comparing the different dosages of Ro 41–5253 in the absence or presence of RAL using one way ANOVA, all *p*<0.05). Results were presented as means ± SD of three independent hepatocyte isolations.

## Results

### Determination of the mRNA levels of enzymes responsible for the retinoids catabolism in primary hepatocytes, and the elevated expression of *Raldh1* and its protein in hepatocytes of ZF rats

Enzymatic processes are involved in the conversions of ROL to RAL and RAL to RA [13], [24], [25]. It has been shown that the short chain dehydrogenase/reductase family 16C member 5 (SDR16C5), RDH2, RDH10, and RALDH1-4 are expressed in the liver [33]. Since RALDH4 is for the biosynthesis of 9-*cis* RA [27], we have focused on the expression levels of the rest enzymes in this study. The *C*_T_ numbers of these genes were normalized to those of 36B4 (an invariable control gene) in the same samples. Based on −ΔC_T_ numbers (how close the abundance of the transcripts of those genes to that of 36B4), we found that *Rdh2*, and *Raldh1* were expressed in meaningful levels (compared with the expression level of 36B4) in both primary rat hepatocytes and HL1C cells (data not shown).

**Figure 3:**
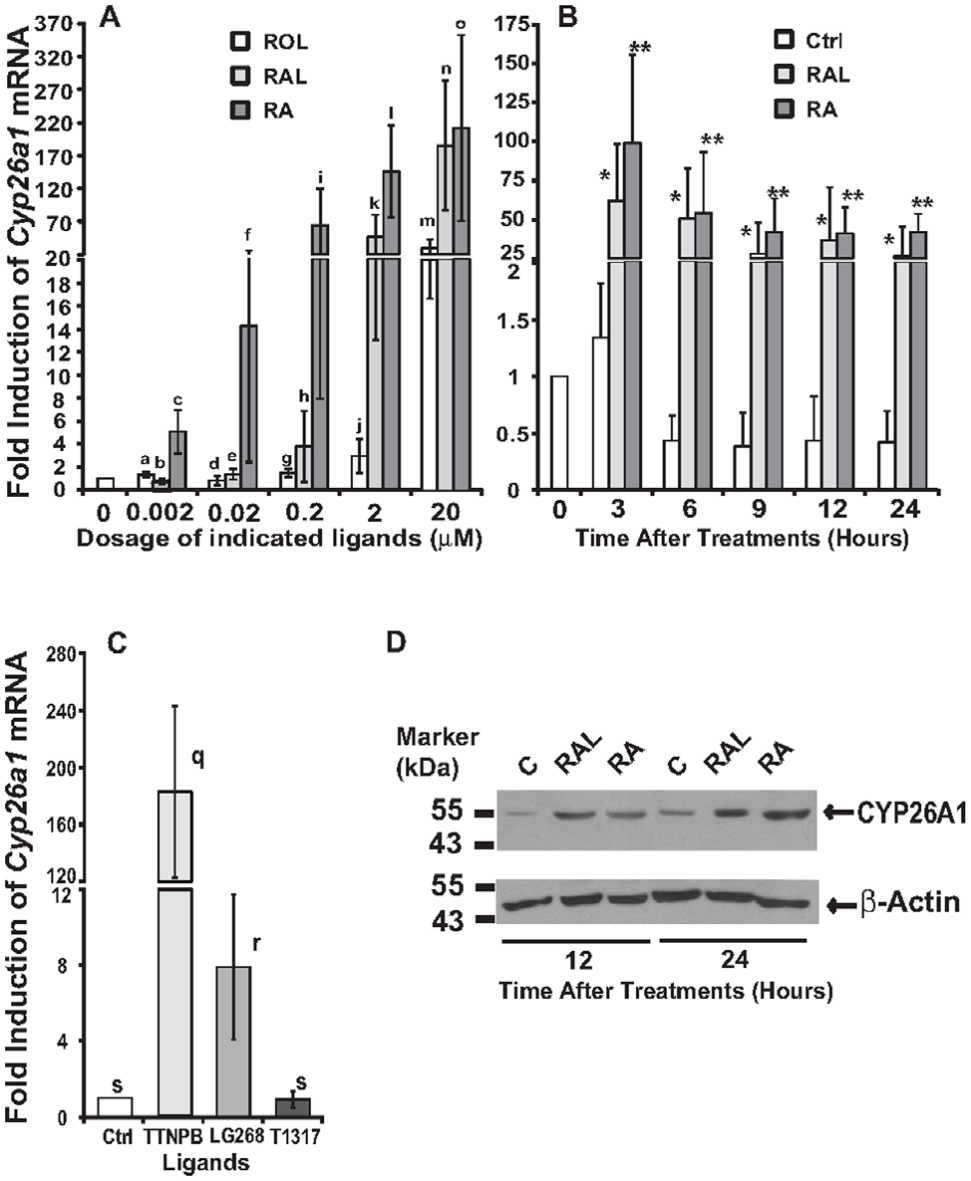
The effects of retinoids on the expression levels of *Cyp26a1* mRNA and CYP26A1 protein in HL1C cells. A. The dose-dependent induction of *Cyp26a1* by ROL, RAL, and RA. HL1C cells were treated with ROL, RAL, or RA at the doses of 0, 0.002 µM, 0.02 µM, 0.2 µM, 2 µM, and 20 µM for 6 h (a/b < c, d/e < f, g/h < i, j < k/l, m < n/o, a/d/g/j < m, b/e/h < k < n, c/f < i/l/o for comparing ROL, RAL and RA at the indicated concentrations, and for comparing each ligand at the different dosages using one way ANOVA; all *p*<0.05). **B.** The expression levels of *Cyp26a1* mRNA overtime (3, 6, 9, 12, and 24 h) in HL1C cells treated with 5 µM RAL or RA (* or ** for comparing RAL or RA with vehicle control groups at the indicated time points using the independent t-test; all *p*<0.05). **C.** The expression levels of *Cyp26a1* mRNA in HL1C cells treated with RAR (TTNPB), RXR (LG268), and LXR (T1317) agonists. HL1C cells were treated with 1 μM TTNPB, LG268, and T1317 for 6 h (q > r>s using one way ANOVA; all *p*<0.05). Total RNA was extracted and subjected to real-time PCR analysis. Data were presented as mean ± SD of three independent treatments. **D.** Whole cell lysates (50 µg/sample) of HL1C cells treated the vehicle control, 5 µM RAL or RA for 12 (lanes 1–3) and 24 (lanes 4–6) hours were separated in 8% SDS PAGE gels, and transferred to the PVDF membranes. Primary antibodies to CYP26A1 (1∶1000 dilution in TBST containing 5% dry milk), and to β-Actin (1∶1000 dilution in TBST containing 5% bovine serum albumin) were recognized by goat anti-rabbit IgG conjugated to horseradish peroxidase, and visualized by chemiluminescence. The films were scanned and presented as described in the Material and Methods.

Since RDH2 catalyzes the conversion of ROL to RAL [33], and RALDH1 is reponsible for converting RAL to RA [33], we compared their expression levels in freshly isolated primary hepatocytes of ZL rats with those of ZF rats. The mRNA level of *Rdh2* in hepatocytes from ZF rats was similar to that from ZL rats. However, the mRNA level of *Raldh1* in hepatocytes from ZF rats was significantly higher than that from ZL rats (Fig. 1A). The results observed in cultured primary hepatocytes of ZL and ZF rats were similar to that in fresh isolated hepatocytes. We observed that the *Rdh2* expression level was unchanged, and the *Raldh1* expression level was elevated significantly in hepatocytes from ZF rats (Fig. 1B). The mRNA level of *Raldh4* was detected, and was at the similar levels in hepatocytes from both ZL and ZF (data not shown).

**Figure 4:**
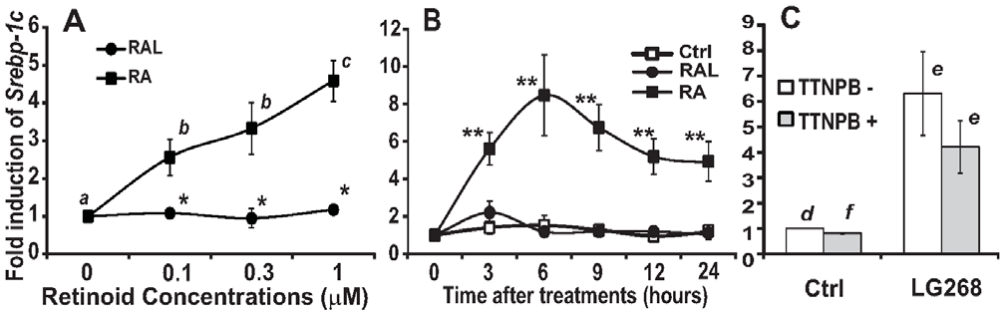
The *Srebp-1c* mRNA levels in INS-1 cells treated with retinoids or ligands. A. 834/40 INS-1 cells were treated with increasing concentrations of RAL or RA for 6 h. **B.** 834/40 cells were treated with 2 µM of RAL or RA for indicated time. **C.** 833/15 cells were treated with 1 µM of specific ligands of RXR (LG268), RAR (TTNPB) or in combination. Total RNA was extracted from INS-1 cells and subjected to real-time PCR analysis. Results were presented as means ± SD of fold inductions (c > b > a for comparing the different dosages of RA-treated groups using one way ANOVA, and e > d > f for comparing control with LG 268 in the absence or presence of TTNPB using one way ANOVA; * for comparing RAL with RA groups at the indicated dosages, and ** for comparing RA with RAL or vehicle control groups at the indicated time points using independent t-test; *n* = 3, all *P*<0.05).

Fig. 1C shows that the RALDH1 protein levels in the liver samples of ZF rats were higher than that of ZL rats. Since two protein bands migrating close to each other were detected in the Immuno-blot, the lysate of INS-1 cells infected by recombinant adenovirus Ad-Raldh1 expressing RALDH1 protein was used as the control to determine the correct size of it. Based on the size of the control sample, the lower band detected by the antibody should be the correct one in the liver samples. Their densities were normalized to the densities of the β-Actin in the same samples. The quantified data were presented as the ratios of RALDH1/β-Actin as shown in Fig. 1C. The ratios of ZF rats are significantly higher than that of ZL rats, demonstrating that the elevation of *Raldh1* mRNA corresponds with an induction of RALDH1 protein.

**Figure 5:**
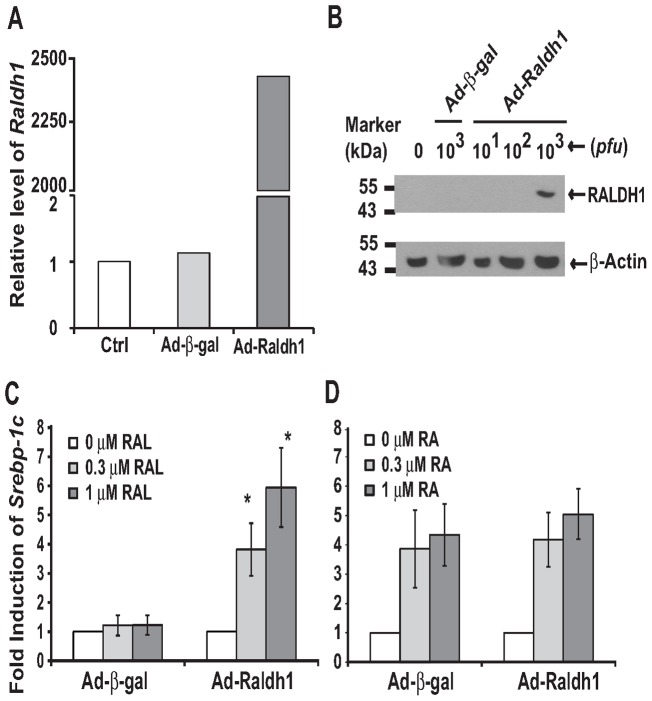
The over-expression of RALDH1 resulted in the RAL-mediated induction of *Srebp-1c* in 833/15 INS-1 cells. A. The adenovirus-mediated *Raldh1* mRNA expression. **B.** Immuno-blot of the over-expression of RALDH1 protein in INS-1 cells. Whole cell lysates (50 µg/sample) of the control cells (lane 1), cells infected by the indicated *pfu* of Ad-β-gal (lane 2) or Ad-Raldh1 (lanes 3–5) were separated in 8% SDS protein gels, and transferred to the PVDF membranes. Primary antibodies to RALDH1 (1∶1000 dilution in TBST containing 5% dry milk), and to β-Actin (1∶1000 dilution in TBST containing 5% bovine serum albumin) were recognized by goat anti-rabbit IgG conjugated to horseradish peroxidase, and visualized by chemiluminescence. The films were scanned and presented as described in the Material and Methods. **C.** RAL only induced *Srebp-1c* expression in cells over-expressing RALDH1, but not β-gal. **D.** RA induced *Srebp-1c* expression in cells over-expressing either β-gal or RALDH1. Results were presented as means ± SD of fold inductions (* for comparing the different dosages of RAL in cells infected by Ad-Raldh1 using one way ANOVA, *n* = 3, all *p*<0.05).

### Retinoids induced the expression levels of *Cyp26a1* mRNA in primary rat hepatocytes

To demonstrate that retinoids catabolism can contribute to the regulation of gene expression in primary rat hepatocytes, the expression levels of *Cyp26a1* mRNA (a RA responsive gene) in response to retinol (ROL) and retinal (RAL) treatments were determined using real-time PCR. Fig. 2A shows a 24-h time course of the expression levels of *Cyp26a1* mRNA in Sprague-Dawley (SD) rat primary hepatocytes treated with 5 µM RA. More than 1000-fold induction of *Cyp26a1* mRNA level was observed at 3 h after the RA treatment, the earliest time point checked. The *Cyp26a1* mRNA level was further induced at 6 h and maintained elevated at 12 h after treatment. At 24 h after the treatment, its level dropped comparing to that at 12 h, but still remained significantly higher than that at time 0.

**Figure 6:**
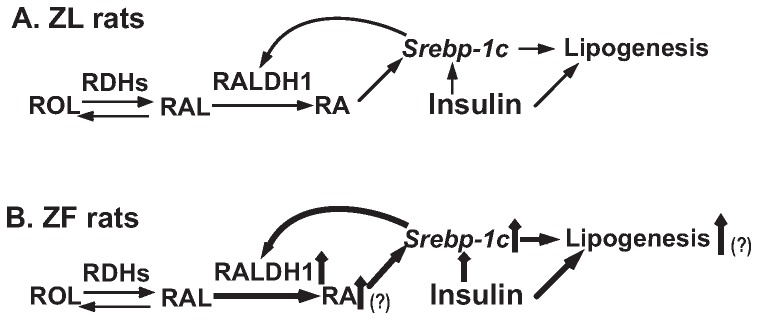
The hypothesized role of the RA production in the feed-forward induction of the expression of *Srebp-1c* and its downstream lipogenic genes in the liver of ZL (A) and ZF (B) rats. **A.** In ZL rats, the hepatic expression of *Srebp-1c* is controlled by the RA production and insulin stimulation. The SREBP-1c also regulates RA production via the induction of *Raldh1* expression until a homeostasis is reached. **B.** The hyperphagia of ZF rats due to the leptin receptor deficiency causes the over-supply of dietary VA, and hyperinsulinemia. The possible elevation of RA production in combination with insulin stimulation leads to higher expression of hepatic *Srebp-1c* in ZF rats, which disrupts the homeostasis. The expression of *Raldh1* mRNA is further induced by SREBP-1c. Therefore, the hepatic expression of *Srebp-1c* in ZF rats is maintained at a higher level than that in ZL rats. The consequence is the elevated hepatic lipogenesis in ZF rats. The up arrows next to the texts indicate induction. The intensified weight of the lines indicates the induction of that part of the pathway. The question marks indicate the steps remained to be confirmed in this hypothesis.

To determine the effects of ROL and RAL on the induction of *Cyp26a1* expression, primary rat hepatocytes from SD rats were treated with increasing concentrations of ROL or RAL for 6 h. As shown in Fig. 2B, the levels of *Cyp26a1* mRNA were induced by ROL and RAL in a dosage dependent manner. ROL and RAL started to induce *Cyp26a1* expression at 2 μM and 0.02 μM, respectively. When the induction folds of *Cyp26a1* expression in response to ROL and RAL treatments were compared, RAL groups had significantly higher values than ROL groups did at concentrations of 0.02 μM, 0.2 μM, 2 μM, and 20 μM. It suggests that RAL is more readily converted to RA than ROL to induce *Cyp26al* expression.

### Inhibition of RAR activation blocked the RAL-mediated induction of *Cyp26a1* expression in primary rat hepatocytes

We have shown that the activation of RAR or/and RXR can induce *Cyp26a1* expression [16]. To confirm the involvement of RAR activation in the RAL-mediated induction of *Cyp26a1* expression, primary SD rat hepatocytes were treated with increasing concentrations of Ro 41–5253, a specific antagonist for RAR activation [34]. As shown in Fig. 2C, RAL at 2 µM dramatically induced the *Cyp26a1* expression, further confirming the result shown in Fig. 2B. This induction was dose-dependently inhibited with increasing concentrations of Ro 41–5253. When the concentration of Ro 41–5253 reached 10 μM or higher, the induction of *Cyp26a1* expression by RAL was completely suppressed. This reverse association between the concentration of Ro 41–5253 and the induction of *Cyp26a1* expression by RAL demonstrated that the inhibition of RAR activation disrupted the retinoid-mediated induction of *Cyp26a1* expression in primary hepatocytes, indicating the important role of RAR activation in this induction.

### Retinoids induced the expression levels of *Cyp26a1* mRNA and CYP26A1 protein in HL1C rat hepatoma cells through the activation of RAR and RXR

To screen for cultured cell lines which we can study the effects of retinoid metabolism on the expression of RA responsive gene, we analyzed the expression of *Cyp26a1* mRNA and CYP26A1 protein in response to ROL, RAL and RA treatments in HL1C rat hepatoma cells [35]. Fig. 3A shows the expression levels of *Cyp26a1* mRNA in HL1C cells treated with the indicated concentrations of retinoids. ROL, RAL and RA started to induce *Cyp26a1* mRNA expression at 20 μM, 2 μM, and 0.2 μM, respectively, demonstrating the different potencies of these retinoids. At 2 μM or 20 μM, the fold induced by RAL was comparable to that by RA. The highest induction fold of *Cyp26a1* mRNA mediated by RA in HL1C cells was near 300-fold which was much lower than that in primary hepatocytes (1000-fold) as shown in Fig. 2. The effects of RAL and RA on *Cyp26a1* mRNA expression in HL1C cells over a 24-h time period were also examined as shown in Fig. 3B. Both RAL and RA induced *Cyp26a1* mRNA expression as early as 3 h. At 6 h after the treatment, the elevated level of *Cyp26a1* mRNA began to drop in comparison to its level at 3 h. At 9, 12, and 24 h time points, its level further declined, but remained significantly higher than that at time 0. The induction was still elevated at 24 h after treatment.

To examine whether the activation of RAR or RXR was sufficient for the induction of *Cyp26a1* mRNA expression, HL1C cells were treated with TTNPB, LG268, and T1317, agonists for RAR, RXR and LXR activation, respectively. As shown in Fig. 3C, the induction folds of the TTNPB, LG268 and T1317 groups are 183.0±60.0, 8.0±3.8, and 1.0±0.4. These results demonstrated that the activation of RAR and RXR, but not LXR, induced the *Cyp26a1* mRNA expression in HL1C cells, indicating that RA activated both RAR and RXR to induce *Cyp26a1* mRNA expression. The induction fold of TTNPB group is much higher than that of LG268 group, similar to that observed in primary hepatocytes [16].

Fig. 3D shows the expression levels of CYP26A1 protein in HL1C cells treated with RAL or RA for 12 and 24 hours. The equal loading of protein samples was confirmed by the similar levels of β-Actin protein among the samples. This result demonstrated that the induction of *Cyp26a1* mRNA resulted in significant elevation of CYP26A1 protein at both time points. All these results suggest that ROL and RAL may be converted to RA, which causes the RAR activation and the subsequent induction of *Cyp26a1* mRNA and CYP26A1 protein levels in HL1C cells.

### RA, but not RAL, induced the *Srebp-1c* expression through the activation of RXR, but not RAR, in INS-1 rat insulinoma cells

We have screened the cultured cell lines in which RAL does not induce *Srebp-1c* expression for a tool to study the conversion of RAL to RA, and shown that RA, but not RAL, induced the *Srebp-1c* expression dose-dependently in 833/15 INS-1 cells [16]. To further confirm that INS-1 cells are an ideal tool to study the conversion of RAL to RA, we analyzed the effects of RAL and RA on *Srebp-1c* expression in 834/40 INS-1 cells, another clonal INS-1 cells [36]. Fig. 4A shows that RA started to significantly induce the *Srebp-1c* expression at 0.1 μM while RAL did not change its expression at any concentration tested. Fig. 4B shows the induction of the *Srebp-1c* expression by RA in 834/40 INS-1 cells over a 24-h time period. RA, but not RAL, induced the *Srebp-1c* expression as early as 3 h and peaked at 6 h. At 9, 12, and 24 h, the induction folds declined in comparison to that at 3 and 6 h, but still maintained significantly higher than that at 0 h. These results and the results shown previously [16] indicate that INS-1 cells may lack the enzymatic activities that convert RAL to RA.

To determine whether the activation of RAR or RXR mediates the effects of RA on the *Srebp-1c* expression, INS-1 cells were treated with TTNPB (1 μM), LG268 (1 μM), or TTNPB + LG268 for 6 h. Fig. 4C shows that LG268 and LG268 + TTNPB induced the *Srebp-1c* expression while TTNPB alone did not induce it. Thus, RXR activation mediates the RA-induced *Srebp-1c* expression in INS-1 cells. All these results demonstrated that the RA-mediated activation of RXR caused the induction of *Srebp-1c* in INS-1 cells, which makes INS-1 cell lines an ideal tool to study the role of RA production in the regulation of lipogenic gene expression.

### The over-expression of RALDH1 resulted in the RAL-mediated induction of *Srebp-1c* in INS-1 cells

To further study the effects of the elevated *Raldh1* mRNA expression in ZF hepatocytes, its full length cDNA was cloned and inserted it into pACCMV5 for the generation of recombinant adenovirus Ad-Raldh1. As shown in Fig. 5, INS-1 cells infected with Ad-Raldh1 had the over-expression of *Raldh1* mRNA (Fig. 5A) and RALDH1 protein (Fig. 5B). The equal loading of the protein samples was confirmed by the similar levels of β-Actin protein among the samples. In INS-1 cells infected with Ad-Raldh1 for overnight (∼18 h), but not in those infected with Ad-β-gal, RAL started to significantly induce the *Srebp-1c* expression at both 0.3 μM and 1 μM (Fig. 5C). On the other hand, RA induced the *Srebp-1c* expression in cells infected by either Ad-β-gal or Ad-Raldh1 (Fig. 5D). In those cells with the over-expression of RALDH1, the induction folds of *Srebp-1c* by RAL treatment were comparable to that by RA. These results indicate that the over-expression of RALDH1 introduced the RAL-mediated induction of *Srebp-1c* in INS-1 cells, indicating the successful conversion of RAL to RA.

## Discussion

In insulin resistant animals, hyperinsulinemia is associated with the elevated hepatic gluconeogenesis and lipogenesis, which should have been respectively suppressed and stimulated by insulin [37]. Our lab has shown that retinoids regulate the expression levels of genes involved in hepatic glucose and fatty acid metabolism. These regulations contributed to the modulation of the insulin-mediated expression of these genes in primary hepatocytes [14]–[16]. The implication of these observations is that the dynamic regulation of RA production may be responsible for the hepatic insulin resistance.

To confirm that the treatments of ROL and RAL can regulate gene expression probably through the production of RA, we analyzed the expression levels of *Cyp26a1*, an RA responsive gene [38], in primary hepatocytes (Fig. 2). Retinoids induced the expression of *Cyp26a1* in a dose- and time-dependent manner. This induction can be blocked in the presence of RAR antagonist, indicating the requirement of RAR activation. This result and the fact that RAL induced the *Cyp26a1* expression indicate that RA production occurs.

To find the cultured cell lines that can be used as tools for further studies of the retinoid catabolism, we have analyzed the retinoid-mediated gene expression in HL1C rat hepatoma cells (Fig. 3). ROL, RAL, and RAL also induced the expression levels of *Cyp26a1* mRNA in HL1C cells in a dose- and time-dependent manner, which is similar to the results obtained in primary hepatocytes. This induction is mediated by the activation of RAR and RXR. The induction of *Cyp26a1* mRNA results in the elevated levels of CYP26A1 proteins in HL1C cells. However, the elevated levels of CYP26A1 protein are not as high as that of *Cyp26a1* mRNA at 12 and 24 hours (Fig. 3B and 3D), indicating the potential roles of protein translation and/or stability in the determination of the CYP26A1 protein levels in HL1C cells. The maximal induction folds by reintoids are higher in primary hepatocytes than in HL1C cells. ROL and RAL respectively induce the expression of *Cyp26a1* mRNA at 2 and 0.02 µM in primary hepatocytes and at 20 and 2 µM in HL1C cells. These results indicate that primary hepatocytes seem to be more readily to convert ROL to RAL and then to RA. Alternatively, HL1C cells are more readily to convert RAL to ROL so that less RA is generated. Whether it is true or not deserves further investigation. It also indicates that HL1C cells can be used as a tool to study the metabolism of retinoids and its effects on the regulation of the expression of RA responsive genes.

It has been shown that the short chain dehydrogenase/reductase family 16C member 5 (SDR16C5), RDH2, RDH10, RALDH1-4 are expressed in the liver [33]. Since *Raldh4* mRNA (encoding RALDH4) is for the production of 9-*cis* RA [27], we focused on *Rdh2* and *Raldh1*. Their mRNA levels in both freshly isolated and cultured primary hepatocytes of ZL and ZF rats were compared. The mRNA level of *Raldh1*, but not that of *Rdh2*, is higher in ZF than that in ZL rat hepatocytes. In addition, the hepatic RALDH1 protein level is also higher in ZF rats than in ZL rats (Fig. 1). The elevated expression levels of *Raldh1* mRNA and RALDH1 protein levels may be resposible for the altered hepatic lipogenesis in ZF rats. To test the hypothesis, we made recombinant adenovirus Ad-Raldh1 to over-express RALDH1 protein in INS-1 rat insulinoma cells. We have shown previously [16] and here (Fig. 4) that RA, but not RAL, induced *Srebp-1c* expression in INS-1 cells. It seems that INS-1 cells lack the enzymatic activities converting RAL to RA. This makes INS-1 cells an ideal tool to test our hypothesis that the elevated expression of RALDH1 in ZF rats causes the increase of RA production, and in turn, the induction of RA responsive gene expression. After the over-expression of *Raldh1* mRNA and RALDH1 protein in INS-1 cells, RAL started to induce the *Srebp-1c* expression as RA did. This indicates that RAL was oxidized to RA by RALDH1 before the induction of *Srebp-1c*. The elevated levels of hepatic *Raldh1* mRNA and RALDH1 protein in ZF rats may contribute to the regulation of hepatic lipogenesis.

It has been shown that the mRNA level of *Raldh1* is elevated in the kidney of *db/db* mice [39]. Both *db/db* mice and ZF rats develop obesity due to the mutation of leptin receptor [4]. It has been shown that ICR mice fed high cholesterol diet have the elevated expression levels of *Raldh1* and *Raldh2*. This is attributed to the oxysterol-induced expression of SREBP-1c which directly binds to the sterol regulatory response elements (SREs) at the proximal promoter regions of *Raldh1* and *Raldh2*, and induces their expression [32]. Since the expression of *Srebp-1c* is induced by insulin [40] and RA [16] in primary hepatocytes, the regulation of *Raldh1* expression becomes a converge point for a feed-forward mechanism by which VA status regulates lipogenesis in the liver. It is reasonable to hypothesiz that in hepatocytes of ZL rats, RA derived from ROL synergizes with insulin to induce the expression of *Srebp-1c* mRNA (Fig. 6A). The mature SREBP-1c protein supports the expression of RALDH1, which promotes the production of RA to maintain the homeostasis of *Srebp-1c* expression in the liver of ZL rats (Fig. 6A). ZF rats are hyperphagic due to the defect of leptin receptor, which leads to the development of obesity, insulin resistance, hyperinsulinemia and hyperlipidemia [41], [42]. We have shown the ZF rat hepatocytes have elevated expression levels of *Srebp-1c* and *Fas* mRNA [43]. We think that the excessive supply of dietary VA caused by hyperphagia and hyperinsulinemia in ZF rats probably work together to trigger an elevation of RA production and an induction of *Srebp-1c* expression in their hepatocytes, respectively. The elevation of the mature SREBP-1c protein induces the expression of *Raldh1* mRNA, which leads to more RA production, and further enhances the expression levels of *Srebp-1c* and its down-stream lipogenic genes. This creates a feed-forward mechanism by which the *Srebp-1c* expression is maintained at a higher level in the liver of ZF rats (Fig. 6B). Whether the proposed feed-forward mechanism is true or not, and what mechanism is responsible for the up-regulation of *Raldh1* expression in the ZF rat liver deserve further investigation.

In summary, the results shown here suggest that ROL and RAL are metabolized into RA to regulate gene expression in rat primary hepatocytes and hepatoma cells. In primary rat hepatocytes, the responsible enzymes are most likely RDH2 and RALDH1. The expression of *Raldh1* mRNA is higher in primary hepatocytes from ZF rats than that from ZL rats, which leads to the elevated RALDH1 protein levels in the liver of ZF rats. Over-expression of RALDH1 introduces the RAL-mediated induction of *Srebp-1c* in INS-1 cells. Thus, we hypothesize that the change of RA production from the over-supply of dietary VA due to the hyperphagia of ZF rats results in higher *Srebp-1c* expression in ZF hepatocytes. The elevated SREBP-1c expression can further induce *Raldh1* expression to create a feed-forward mechanism that could be one of the reasons responsible for the increased lipogenesis in the liver of ZF rats.

## Materials and Methods

### Reagents

The reagents for primary hepatocyte isolation and culture have been published previously [44]. Source of LG268 was reported previously as well [15]. All other compounds were purchased from Sigma (Saint Louis, MO) unless described otherwise. The reagents for cDNA synthesis and real-time PCR were obtained from Applied Biosystems (Foster city, CA). Medium 199, liver perfusion medium and liver digest buffer were obtained from Invitrogen (now Life Technologies, Grand Island, NY 14072). Dulbecco's Modification of Eagle Medium (DMEM), RPI-1640 medium, fetal bovine serum, antibiotics, and trypsin-EDTA solution were obtained from Fisher Scientific (Pittsburgh, PA 15275).

### Animals

Male Sprague-Dawley (SD) rats were purchased from Harlan Breeders (Indianapolis, IN). Male Zucker lean (ZL) and ZF rats were bred at UTK. Rats were housed in colony cages, and fed a standard rodent diet before isolation of primary hepatocytes. All procedures were approved by the Institutional Animal Care and Use Committee at the University of Tennessee at Knoxville (Protocol numbers 1642 and 1582).

### Cultures of primary rat hepatocytes, HL1C rat hepatoma cells, 293 human embryonic kidney cells, and INS-1 rat insulinoma cells

Methods for the primary rat hepatocyte preparation and analysis of RNA were described previously [44]. For the hepatocyte isolation and pretreatment, the rat was euthanized with carbon dioxide. A catheter was inserted into portal vein and connected to a peristaltic pump with liver perfusion medium and liver digestive buffer. The inferior vena cava was cut open to allow the outflow of the media at 10 ml/minute. After completion of the digestion, the liver was excised and put into a tissue culture dish containing liver digest buffer for removing the connection tissues and allowing the release of hepatocytes. The medium containing hepatocytes was filtered through a cell strainer (100 µm), and precipitated by 50× g centrifugation for 3 minutes (min). The cell pellet was washed twice with DMEM containing 5% fetal bovine serum (FBS), 100 U/ml sodium penicillin, and 100 µg/ml streptomycin sulfate after re-suspension and precipitation. After the wash, the isolated hepatocytes were plated onto 60-mm collagen type I coated dishes at 3×10^6^ cells/ per dish and incubated in 4 ml of the same medium at 37°C and 5% CO2 for 3 to 4 hours (h). The attached cells were washed once with 4 ml of PBS, and incubated in medium A (Medium 199 supplemented with 100 nM dexamethasone, 100 nM 3, 3′, 5-triiodo-l-thyronine (T3), 100 units/ml penicillin, and 100 μg/ml streptomycin sulfate) containing 1 nM insulin for overnight (14–16 h) until being used for the indicated experiments.

The HL1C cells [35] which was stably transfected with a reporter gene construct containing a fragment of *Pck1* promoter sequence were kindly provided by Dr. Donald K. Scott at University of Pittsburgh. They were maintained in DMEM containing 4.5 g/L glucose, 4% FBS, 100 U/ml of penicillin and 100 μg/ml streptomycin sulfate. They were incubated in 60 mm dishes with serum free DMEM containing the indicated reagents shown in the figure legends for the indicated length of time before being subjected to analysis.

The 833/15 and 834/40 clonal insulinoma INS-1 cells [36] were cultured in medium B (RPMI 1640, 2 mM L-glutamine, 1 mM Na-pyruvate, 50 μM β-mercaptoethanol, 100 U/ml of penicillin and 100 μg/ml streptomycin sulfate) containing 10% FBS as described previously. For the treatment, INS-1 cells in 60 mm dishes were incubated in medium B containing the indicated reagents shown in the figure legends for the indicated length of time before being subjected to analysis.

The HEK 293 cells were cultured in DMEM containing 4.5 g/L glucose, 8% FBS, 100 U/ml of penicillin and 100 μg/ml streptomycin sulfate at 37°C and 5% CO_2_.

### RNA extraction and quantitative real-time PCR

Total RNA was extracted from the treated cells on one 60 mm dish using 1 ml of RNA STAT 60 reagent (TEL-TEST, Inc, Friendswood, TX) according to the manufacture's protocol. The contaminated DNA was removed using the DNA-*free* ^TM^ kit (Applied Biosystems). First strand cDNA was synthesized from 2 µg of DNA-free RNA with random hexamer primers using cDNA synthesis kit (Applied Biosystems). The real-time PCR primer sequences for detecting *Cyp26a1* [45], *Srebp-1c* [16], and *Raldh1* [46] have been reported previously. The primers for detecting *Rdh2* (sense 5′-CAAGTTCTTCTACCTCCCCATGA-3′, and anti-sense 5′-TCCAGTAGAAAAGGGCATCCA-3′) were designed using software Primer Express 3.0 (Applied Biosystems). Each SYBR green based real-time PCR reaction contains, in a final volume of 14 µl, cDNA from 14 ng of reverse transcribed total RNA, 2.33 pmol primers, and 7 µl of 2× SYBR Green PCR Master Mix (Applied Biosystems). Triplicate PCR reactions were carried out in 96-well plates using 7300 Real-Time PCR System. The conditions are 50°C for 2 min, 95°C for 10 min, followed by 40 cycles of 95°C for 15 seconds (s) and 60°C for 1 min. The gene expression level was normalized to that of invariable control gene, *36B4*. Data are presented as either minus Δ cycle threshold *C*_T_ [43] or the induction fold for which the control group is arbitrarily assigned a value of 1 using ΔΔ*C*_T_ method as previously described [44].

### Cloning of the rat *Raldh1* cDNA and generating Ad-Raldh1 recombinant adenovirus for the over-expression of RALDH1

To clone the rat *Raldh1* cNDA, sense 5′-AGCCAAACCAGCAATGTCTTC-3′, and anti-sense 5′-CACTCTGCTTCTTAGGAGTTC-3′ primers were designed based on the its mRNA sequence (reference number NM_022407.3) from the National Center for Biotechnology Information. The *Raldh1* cDNA containing the entire coding sequence was amplified using SD rat primary hepatocyte cDNA as a template. The amplicon was ligated into pCR®2.1 vector through TA Cloning® Kit (Invitrogen) and processed according to the manufacture's protocol. The full length cDNA with the correct sequence confirmed by DNA sequencing was inserted to pACCMV5 for making pACCMV5-Raldh1.

To generate Ad-Raldh1 recombinant adenovirus, plasmids pACCMV5-Raldh1 and JM17 were co-transfected into 293 cells using FuGENE®6 Transfection Reagent (Roche Applied Science, Indianapolis, IN). Transfected 293 cells were incubated in DMEM containing 2% FBS at 37°C and 5% CO_2_ until the formation of the plaques due to the lysis of the cells. The crude lyaste was collected and stored at −80°C. The presence of *Raldh1* cDNA sequence in the viral DNA was confirmed by PCR.

### Preparation and purification of recombinant adenoviruses

To generate the crude lysate, HEK 293 cells grew in 100 mm tissue culture plates at 80% confluence were infected by the original crude lysate containing Ad-Raldh1. The ratio of the medium to crude lysate is 10 to 1. After the lysis of the cells at around 48 h post infection, the cell culture medium (the crude lysate) was collected, and stored at −80°C until being used.

For purification of the recombinant adenovirus, NP-40 was first added into the crude lysate to reach the final concentration at 0.5%. The mixture was shaken gently at room temperature for 30 min and subjected to centrifugation at 8,000 rpm and 4°C for 15 min. The supernatant was transferred to a clean bottle, and 0.5× volume of 20% PEG8000/2.5 M NaCl was added. The preparation was shaken gently at 4°C overnight. The resulting mixture was transferred to centrifuge bottles and spun at 13,000 rpm at 4°C for 15 min. The precipitated pellet was re-suspended in a small volume of PBS (2–3 ml), and spun at 8,000 rpm and 4°C for 10 min to remove insoluble matters. Solid CsCl was added to the supernatant until its final density reached 1.34 g/ml. The mixture was spun at 90,000 rpm and 25°C for 3 h using Optima^TM^ MAX-XP Ultracentrifuge (Beckman Coulter, Inc.). The corresponding band containing pure viral particles was collected in a total volume less than 1 ml for desalting. The PD-10 column Sephadex^TM^ G-25 M (Amersham Pharmacia Biotech AB, Sweden) was equilibrated with 5 ml PBS. The purified virus in CsCl solution was loaded onto the column and eluted with 5 ml PBS. The flow through was collected into ten fractions. The optical densities (OD) at 260–nm of these fractions were determined using Spectronic® GENESYS^TM^ 5 Spectrophotometer (Thermo Scientific). The fractions containing significant values of absorbance (usually at around fractions 7–9) were collected and pooled. Bovine serum albumin and glycerol were added to the pooled solution to make the stock viral solution with the final concentrations of them at 0.2% and 10%, respectively. After the filtration of the stock solution for sterilization, its OD was determined to estimate the plaque forming units (*pfu*). We used that 1 OD equals to 1×10^12^ *pfu*/ml. The final purified virus stock was frozen at −80°C until being used in the indicated experiments.

### Immuno-blot and quantification of the protein bands

INS-1 cells were infected with purified Ad-Raldh1 for 24 h. The cells in 60 mm dishes were washed once with 3 ml PBS and scrapped from the dish into 400 μl of whole-cell lysis buffer (1% Triton X-100, 10% glycerol, 1% IGEPAL CA-630, 50 mM Hepes, 100 mM NaF, 10 mM EDTA, 1 mM sodium molybdate, 1 mM sodium β-glycerophsphate, 5 mM sodium orthovanadate, 1.9 mg/ml aprotinin, 5 μg/ml leupeptin, 1 mM benzamide, 2.5 mM PMSF, pH 8.0). The lysates were placed on ice for at least 20 min before they were subjected to centrifugation at 13,000 rpm for 20 min. The protein concentration in the supernatant was determined with PIERCE BCA protein assay kit (Rockford, IL). Proteins (40 μg/lane) in whole cell lysates were separated on SDS-PAGE, transferred to BIO-RAD Immuno-Blot PVDF membrane (Hercules, CA) and detected with primary antibodies to RALDH1 (for Fig. 5, catalog #2052-1, Epitomics, Burlingame, CA 94010), RALDH1 (for Fig. 1, catalog #AP1465a, Abgent, San Diego, CA 92121), CYP26A1 (catalog # CYP26A11-A, Alpha Diagnostics International, TX 78244), and β-Actin (#4970 s, Cell Signaling Technology, Danvers, MA 01923) according to the protocols provided by the manufacturers. Bound primary antibodies were visualized by chemiluminescence (ECL Western Blotting Substrate, Thermo Scientific) using a 1∶2,000 dilution of goat anti-rabbit IgG (#7074P2, Cell signaling Technology) conjugated to horseradish peroxidase. Membranes were exposed to X-ray films (Phenix Research Products, Candler, NC) for protein band detection. The films were scanned using an HP Scanjet 3970 (Palo Alto, CA 94304) and stored as Tagged Image File Format (TIFF). The ImageJ Software (http://rsbweb.nih.gov/ij/) was used to determine the density of a protein band by subtracting the background in an area of the same size immediately in front of protein band. The ratio of the densities of RALDH1 and β-Actin in the same sample was calculated and used for statistical analysis.

### Statistics

Data are presented as means ± S.D. The number of experiments represents the independent experiments using hepatocytes isolated from different animals or indicated cells cultured in different days. Levene's test was used to determined homogeneity of variance among groups using SPSS 19.0 statistical software and where necessary natural log transformation was performed before analysis. An independent-samples t-test was used to compare two conditions. Multiple comparisons were analyzed by one-way analysis of variance (ANOVA) using least significant different (LSD) when equal variance was assumed, and Games-Howell test was used when equal variance was not assumed. Differences were considered statistically significant at *P*<0.05.
